# The Influence of *Nrf2* on Cardiac Responses to Environmental Stressors

**DOI:** 10.1155/2013/901239

**Published:** 2013-04-22

**Authors:** Reuben Howden, Eva Gougian, Marcus Lawrence, Samantha Cividanes, Wesley Gladwell, Laura Miller-DeGraff, Page H. Myers, D. Clay Rouse, Robert B. Devlin, Hye-Youn Cho, Steven R. Kleeberger

**Affiliations:** ^1^Laboratory of Systems Physiology, Department of Kinesiology, University of North Carolina at Charlotte, Charlotte, NC, USA; ^2^Laboratory of Respiratory Biology, National Institute of Environmental Health Sciences, National Institutes of Health, Research Triangle Park, NC, USA; ^3^Comparative Medicine Branch, National Institute of Environmental Health Sciences, National Institutes of Health, Research Triangle Park, NC, USA; ^4^Division of Laboratory Animal Resources, Duke University Medical Center, Durham, NC, USA; ^5^United States Environmental Protection Agency, Research Triangle Park, NC, USA

## Abstract

*Nrf2* protects the lung from adverse responses to oxidants, including 100% oxygen (hyperoxia) and airborne pollutants like particulate matter (PM) exposure, but the role of *Nrf2* on heart rate (HR) and heart rate variability (HRV) responses is not known. We hypothesized that genetic disruption of *Nrf2* would exacerbate murine HR and HRV responses to severe hyperoxia or moderate PM exposures. *Nrf*2^−/−^ and *Nrf*2^+/+^ mice were instrumented for continuous ECG recording to calculate HR and HRV (low frequency (LF), high frequency (HF), and total power (TP)). Mice were then either exposed to hyperoxia for up to 72 hrs or aspirated with ultrafine PM (UF-PM). Compared to respective controls, UF-PM induced significantly greater effects on HR (*P* < 0.001) and HF HRV (*P* < 0.001) in *Nrf*2^−/−^ mice compared to *Nrf*2^+/+^ mice. *Nrf*2^−/−^ mice tolerated hyperoxia significantly less than *Nrf*2^+/+^ mice (~22 hrs; *P* < 0.001). Reductions in HR, LF, HF, and TP HRV were also significantly greater in *Nrf*2^−/−^ compared to *Nrf*2^+/+^ mice (*P* < 0.01). Results demonstrate that *Nrf2* deletion increases susceptibility to change in HR and HRV responses to environmental stressors and suggest potential therapeutic strategies to prevent cardiovascular alterations.

## 1. Introduction

The deleterious effects of environmental exposures and associated oxidative stress on the cardiopulmonary system are well established and present one of the most significant public health problems [1]. Diseases and disorders of the cardiopulmonary system associated with an enhanced oxidant load include, but are not limited to, inflammatory lung diseases (e.g., acute respiratory distress syndrome [2] and bronchopulmonary dysplasia [3, 4]) and a host of cardiovascular (CV) diseases (e.g., atherosclerosis [5, 6], hypertension [7], and heart failure [8]).

 Exposure to oxidants can exacerbate the pathogenesis of these diseases by further increasing oxidative stress and in some cases overwhelm antioxidant defenses. Inflammatory lung disease and post-resuscitation from cardiac arrest are frequently treated with oxygen therapy (hyperoxia), which can cause significant lung injury [9], adverse cardiac responses [10], and death if exposure is sufficiently long, even in young healthy laboratory animals.

However, not all oxidants such as air pollution produce overt outcomes, but they are no less problematic in terms of public health because exposure is frequent, wide spread, and exacerbated by other influential factors such as age and preexisting disease. One prominent example is exposure to particulate matter (PM). PM is a diverse composition of metals and inorganic matter, the constituents of which are dependent on the source, geographic region, and particle aerodynamic diameter which have been reviewed in detail [11]. Exposure to PM is known to induce pulmonary [12–14] and cardiovascular [15, 16] responses, which have been associated with increases in hospital admissions and premature mortality (for review [17]), especially in those with preexisting cardiopulmonary disease. Direct and indirect pathways for PM-induced effects on cardiovascular function have been proposed ([18, 19] for review). Indirect effects include lung exposure derived influences on the cardiovascular system via alterations in nervous system function [20, 21], thus altering heart rate variability (HRV) [22–24] and systemic [25] and/or vascular inflammation [26]. Direct PM effects on cardiovascular function have been associated with infiltration of PM, especially PM with an aerodynamic diameter of <0.1 *μ*m (UF-PM) [27, 28]. Subsequent effects include vascular dysfunction [29, 30] and increased oxidant burden [31–33].

Resistance to oxidant stress relies upon effective antioxidant defenses including enzymes NAD(P)H:quinone oxidoreductase 1 (NQO1), superoxide dismutase (SOD), glutathione peroxidases, and heme oxygenase-1 (HO-1). These and other phase II enzyme genes contain promoter antioxidant response elements (AREs) which bind to a heterodimer containing a small Maf protein and nuclear factor-erythroid 2-(NF-E2-) related factor 2-(*Nrf2*), a member of the Cap “n” Collar family of transcription factors. Although the role of *Nrf2* in cardiovascular diseases is complex (refer to a review by R. Howden in the current issue), it has been implicated in resistance against lung injury induced by oxidant exposure [34–36].

Recently, significant adverse changes in cardiac function were reported in mice during exposure to hyperoxia [10], a well-established murine model for acute lung injury and inflammatory lung disease [37, 38], and results suggested a genetic component to cardiac responses. Furthermore, several studies have reported cardiovascular responses to PM exposure, especially heart rate variability (HRV), but genetic factors leading to susceptibility are poorly defined (for review [39]).

Changes in HR and HRV are accepted as indicators for increases in cardiovascular risk, including in response to oxidative stress [10, 40–42]. The purpose of this study was to test the hypothesis that *Nrf2* protects against the cardiac responses (HR and HRV) to hyperoxia or UF-PM exposure and improve understanding of the widespread importance of *Nrf2* activity in resistance to oxidative stress.

## 2. Materials and Methods

### 2.1. Animals and Survival Surgery

Male ICR/sv129: *Nrf*2^−/−^ and ICR/sv129: *Nrf*2^+/+^ (wild-type littermates) mice were obtained from a colony maintained at NIEHS, and were originally developed at Tsukuba University [43]. Mice *n* = 8 − 16 (per strain; 20–30 g; 8–12 weeks of age) were housed individually in standard polycarbonate cages with a 12 : 12 hours light-dark cycle. Food (AIN-76A) and water were provided *ad libitum.* Animals were handled in accordance with The National Institutes of Health Humane Care and Use of Laboratory Animals guidelines. The study protocol was reviewed and approved by the National Institute of Environmental Health Science Animal Care and Use Committee.

Mice were anesthetized with inhaled isoflurane (1.5–2% in oxygen) with buprenorphine (0.1 mg/Kg) given for analgesia. Following a midline dorsal cutaneous incision (3 cm), a subcutaneous tissue pocket was made with a blunt instrument, into which an ETA-F20 ECG transmitter (DSI; Arden Hills, MN, USA) was placed. The positive and negative ECG leads were sutured over the left superficial gluteus and right trapezius muscles, respectively. All incisions were closed using wound clips and animals recovered for five days.

### 2.2. Hyperoxia and Ultrafine Particulate Matter (UF-PM) Exposure

Prior to any exposure, mice were housed in individual whole body plethysmographs (Buxco Electronics, Wilmington, NC, USA) and allowed at least 30 minutes to become quiescent before recording 20 minutes of baseline ECG. Mice of each genotype were randomly assigned to the following groups: group 1, UF-PM exposure by aspiration (*n* = 4 per strain; normoxia); group 2, saline exposure by aspiration under normoxic conditions (*n* = 4 per strain; normoxia); and group 3, hyperoxia exposure (*n* = 8 per strain; no UF-PM or saline exposure). The number of mice exposed to UF-PM (group 1) was lower because the particles were in limited supply.

UF-PM (aerodynamic diameter <0.1 *μ*m) was collected at the University of North Carolina at Chapel Hill in 2002 [44]. Mice were anesthetized by isoflurane and exposed by aspiration to 100 *μ*g UF-PM suspended in 50 *μ*L of sterile 0.9% saline (group 1) or 50 *μ*L of sterile 0.9% saline only (group 2). Saline/UF-PM suspension was vortexed immediately prior to dosing each animal. Within 10–15 min of UF-PM exposure, mice were housed in whole body plethysmographs (for consistency with hyperoxia exposure procedures below) for 48 hr of continuous ECG data recording.

Group 3 mice were exposed to 100% oxygen using individual whole body plethysmographs as exposure chambers. The oxygen was delivered from a liquid oxygen tank, warmed to room temperature, and sufficiently humidified. ECG was recorded continuously, while mice were exposed to hyperoxia for a maximum of 72 hr, until moribund or when HR declined to ~250 bpm. These endpoints were chosen based on previous studies of prolonged hyperoxia exposure of inbred mice [10]. 

R-R interval and HR data were calculated from the ECG records using specialist ECG pattern recognition software (Ponemah, v4.8-SP4). We calculated HRV using a Lomb periodogram as described previously [45]. The frequency ranges used were 0.2–1.5 Hz (low frequency; LF) and 1.5–50 Hz (high frequency; HF), and a summation of the LF and HF was used to represent total power (TP).

### 2.3. Statistical Analysis

Group mean baseline phenotypic values (HR and HRV) for each genotype were calculated, and differences were assessed independently using a one-way ANOVA (alpha level was set at 0.05). HR and HRV responses (hourly means ± SEM) to hyperoxia (genotype X time) were assessed independently using a two-way ANOVA with SNK post hoc test for pairwise comparisons up to the common length of exposure time between *Nrf*2^−/−^ and *Nrf*2^+/+^ groups (45 hr exposure; alpha level was set at 0.05). Differences in HR and HRV phenotypes between baseline and hyperoxia (hourly means ± SEM) were assessed for each genotype using a one-way ANOVA (alpha level was set at 0.05). To assess changes in HR and HRV in response to hyperoxia in the *Nrf*2^+/+^ mice (i.e., a longer exposure time compared to *Nrf*2^−/−^) from baseline, a one-way ANOVA (*P* < 0.05) was used. HR and HRV responses to UF-PM exposure (genotype X treatment X time) were assessed independently using a three-way ANOVA with Students-Newman-Keuls post hoc test for pairwise comparisons (alpha level was set at 0.05). 

## 3. Results

### 3.1. HR and HRV Responses to UF-PM

**Figure 1:**
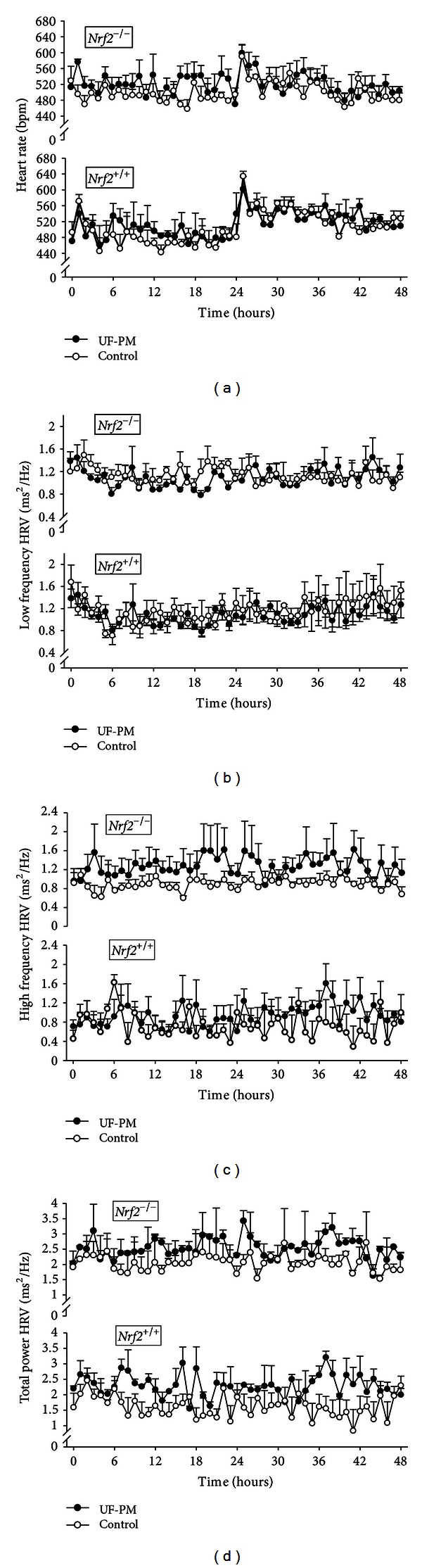
(a) Heart rate (HR, bpm) responses in *Nrf*2^−/−^ and *Nrf*2^+/+^ mice following aspiration of either ultrafine particulate matter (UF-PM, <0.1 *μ*m) in saline or saline alone. Significant overall effects between treatment and genotype were found (*P* < 0.05; Table 1). (b) Low frequency heart rate variability (HRV, ms^2^/Hz) responses in *Nrf*2^−/−^ and *Nrf*2^+/+^ mice following aspiration of either ultrafine particulate matter (UF-PM, <0.1 *μ*m) in saline or saline alone. Significant overall effects between treatments only were found (*P* < 0.05; Table 1). (c) High frequency (HF) heart rate variability (HRV, ms^2^/Hz) responses in *Nrf*2^−/−^ and *Nrf*2^+/+^ mice following aspiration of either ultrafine particulate matter (UF-PM, <0.1 *μ*m) in saline or saline alone. Significant overall effects between treatment and genotype were found (*P* < 0.05; Table 1). (d) Low frequency (LF) heart rate variability (HRV, ms^2^/Hz) responses in *Nrf*2^−/−^ and *Nrf*2^+/+^ mice following aspiration of either ultrafine particulate matter (UF-PM, <0.1 *μ*m) in saline or saline alone. Significant overall effects between treatment and genotype were found (*P* < 0.05; Table 1). Group means ± SEM are presented (*n* = 4/group).

No significant differences in group mean baseline HR, LF, HF, or TP were detected between *Nrf*2^−/−^ and *Nrf*2^+/+^ (519.8 ± 18.3 versus 482.8 ± 13.9 bpm; 1.14 ± 0.17 versus 1.51 ± 0.16 ms^2^/Hz; 0.94 ± 0.17 versus 0.75 ± 0.09 ms^2^/Hz; and 2.08 ± 0.18 versus 2.26 ± 0.22 ms^2^/Hz, resp.; Figures 1(a)–1(d)). However, compared to saline, a significant overall increased effect of UF-PM exposure on HR responses was found in *Nrf*2^−/−^ mice (48 hr mean difference 21.26 bpm; *P* < 0.001; Figure 1(a) and Table 1) but not in *Nrf*2^+/+^ mice. Moreover, HR responses were significantly greater in *Nrf*2^−/−^ compared to *Nrf*2^+/+^ mice treated with UF-PM (48 hr mean difference 24.26 bpm; *P* < 0.001).

A significant overall reduction effect of UF-PM treatment on LF HRV responses (48 hr mean difference 0.02 ms^2^/Hz; *P* = 0.048; Figure 1(b) and Table 1) was also found, but it was not dependent on genotype. However, multiple significant effects on HF HRV were detected (Figure 1(c) and Table 1). Interestingly, an overall significantly increased HF HRV was found within *Nrf*2^−/−^ mice treated with UF-PM versus saline (48 hr mean difference 0.37 ms^2^/Hz; *P* < 0.001), but not within *Nrf*2^+/+^ mice (Table 1). Moreover, overall genotype effects were found for saline and UF-PM treatment groups (48 hr mean difference 0.15 ms^2^/Hz; *P* = 0.019 and 48 hr mean difference 0.33 ms^2^/Hz; *P* < 0.001, resp.; Table 1). However, it is important to note that the studentized range distribution (*q* value) was more than three times higher when comparing *Nrf*2^+/+^ and *Nrf*2^−/−^ treatment groups (*q* = 2.40 and 9.04, resp.) and more than twice as high for *Nrf*2^+/+^ versus *Nrf*2^−/−^ within UF-PM or saline (*q* = 3.32 and 8.06 resp.; Table 1), suggesting a greater effect in *Nrf*2^−/−^ mice when treated with UF-PM. Moreover, the interactions for HF HRV between genotypes within each treatment group were opposing. Overall, HF HRV was higher in *Nrf*2^+/+^ versus *Nrf*2^−/−^ mice following saline treatment, but HF HRV was higher in *Nrf*2^−/−^ versus *Nrf*2^+/+^ mice following UF-PM treatment (Table 1). Specific time points at which these differences occurred were undetectable, perhaps due to the high degree of variability in the *Nrf*2^−/−^ UF-PM treated group. 

TP HRV is the sum of HF and LF HRV, and multiple overall effects were found (Figure 1(d) and Table 1), but specific time points at which these differences occurred were not detectable. Overall differences in TP HRV responses between UF-PM and saline treatment were found within *Nrf*2^+/+^ and *Nrf*2^−/−^ groups (48 hr mean difference for *Nrf*2^+/+^, 0.17 ms^2^/Hz, *P* = 0.007; 48 hr mean difference for *Nrf*2^−/−^, 0.35 ms^2^/Hz, *P* < 0.001). Moreover, *Nrf*2^−/−^ and *Nrf*2^+/+^ groups were different from each other with respect to TP HRV responses irrespective of treatment (48 hr mean difference after saline, 0.19 ms^2^/Hz, *P* = 0.004; 48 hr mean difference after UF-PM, 0.34 ms^2^/Hz, *P* < 0.001). However, the *q* value was approximately twice as high when comparing *Nrf*2^+/+^ and *Nrf*2^−/−^ treatment groups (*q* = 3.78 and 8.22, resp.) and *Nrf*2^+/+^ and *Nrf*2^−/−^ within UF-PM or saline (*q* = 4.06 and 7.93, resp.), again suggesting a greater effect in *Nrf*2^−/−^ mice treated with UF-PM, although this effect was primarily influenced by HF HRV responses as the interactions were similar (Table 1). Despite the significant differences in HR and HRV responses between treatment and genotype, we were not able to detect specific posttreatment time points where these differences lie.

### 3.2. HR and HRV Responses to Hyperoxia

**Figure 2:**
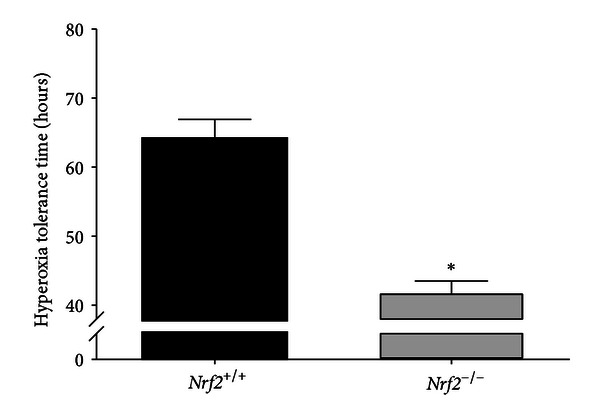
Time to heart rates of 250 bpm in *Nrf*2^−/−^ and *Nrf*2^+/+^ mice during hyperoxia (100% oxygen) exposure. *Significantly different between genotypes (*P* < 0.001). Group means ± SEM are presented (*n* = 8/group).

In mice used for hyperoxia exposures, no significant differences in group mean (±SEM) baseline HR, LF, HF, or TP were detected between *Nrf*2^−/−^ and *Nrf*2^+/+^ (484.2 ± 21.2 versus 486.6 ± 12.3 bpm; 1.17 ± 0.18 versus 1.35 ± 0.10 ms^2^/Hz; 1.25 ± 0.18 versus 1.12 ± 0.14 ms^2^/Hz; 2.42 ± 0.17 versus 2.47 ± 0.17 ms^2^/Hz, resp.; Figure 2). 

**Figure 3:**
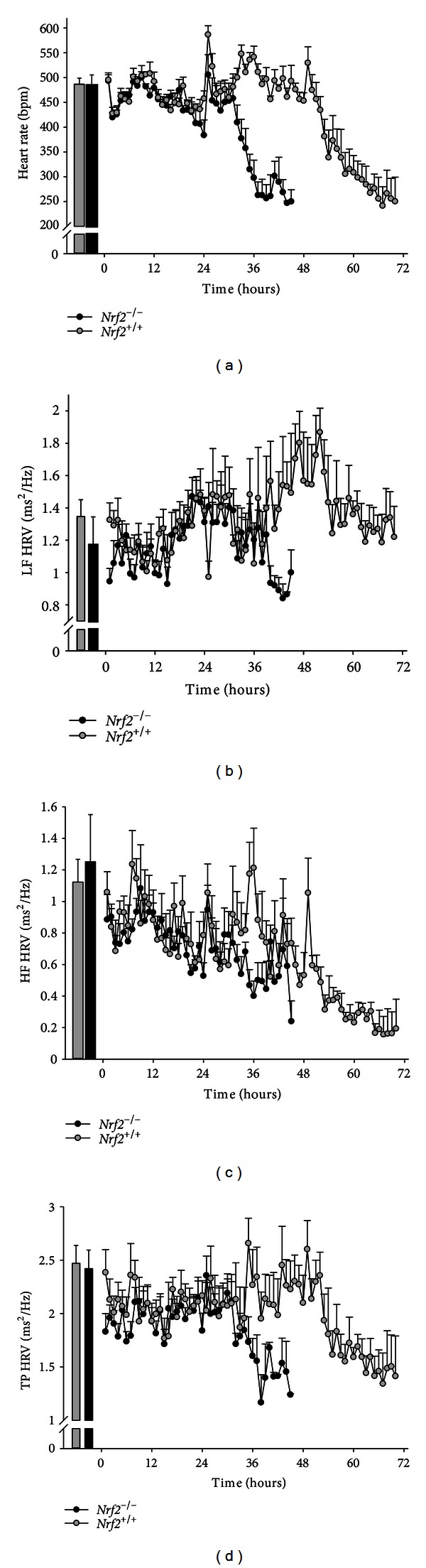
(a) Hourly mean heart rate (HR, bpm) responses in *Nrf*2^−/−^ and *Nrf*2^+/+^ mice during hyperoxia (100% oxygen) exposure. Heart rates in *Nrf*2^−/−^ mice were significantly reduced from baseline from 34 hr until the end of exposure (*P* < 0.05). Heart rates in *Nrf*2^+/+^ mice were significantly reduced from baseline from 54 hr until the end of exposure (*P* < 0.05). (b) Hourly mean low frequency (LF) heart rate variability (HRV, (ms^2^/Hz) responses in *Nrf*2^−/−^ and *Nrf*2^+/+^ during hyperoxia (100% oxygen) exposure. LF HRV reduced in *Nrf*2^−/−^ *versus* *Nrf*2^+/+^ after 40 hr of exposure (*P* < 0.05). LF HRV in *Nrf*2^+/+^ mice did not change significantly (*P* > 0.05). (c) Hourly mean high frequency (HF) heart rate variability (HRV, ms^2^/Hz) responses in *Nrf*2^−/−^ and *Nrf*2^+/+^ during hyperoxia (100% oxygen) exposure. HF HRV reduced significantly in *Nrf*2^−/−^ *versus* *Nrf*2^+/+^ at 35 and 36 hrs of exposure (*P* < 0.05). (d) Hourly mean total power heart rate variability (TP HRV, ms^2^/Hz) responses in *Nrf*2^−/−^ and *Nrf*2^+/+^ during hyperoxia (100% oxygen) exposure. TP HRV reduced in *Nrf*2^−/−^ *versus* *Nrf*2^+/+^ after 43 hr to the end of the exposure (*P* < 0.05). Group means ± SEM are presented (*n* = 8/group).

 Group mean (±SEM) HR of *Nrf*2^−/−^ mice reduced to below 250 bpm in significantly less time compared to *Nrf*2^+/+^ mice (determined by the first hr at which individual mouse HR was less than 250 bpm was detected; 41.6 ± 1.9 versus 64.0 ± 2.9 hours; *P* < 0.001; Figure 2). Prolonged hyperoxia caused highly significant and precipitous reductions in HR after a period of normal circadian variation, which was genotype dependent. Compared to respective genotype mean baseline values, HR reduced significantly in *Nrf*2^−/−^ mice after 34 hrs hyperoxia (group mean difference 178.2 bpm; *P* < 0.001) and continued to decline until exposure terminated at 45 hrs (group mean difference 234.4 bpm; *P* < 0.001; Figure 3(a)). In *Nrf*2^+/+^ mice, the decline in HR compared to baseline was not significant until 54 hrs hyperoxia (group mean difference 147.8 bpm; *P* < 0.001; Figure 3) and 20 hrs after a significant group mean HR reduction in *Nrf*2^−/−^ mice. HR continued to decline in *Nrf*2^+/+^ mice until exposure terminated at 70 hr (group mean difference 236.4 bpm; *P* < 0.001; Figure 3(a)).

 LF HRV was significantly reduced in *Nrf*2^−/−^ mice compared to *Nrf*2^+/+^ mice after 40 hrs hyperoxia (group mean difference 0.63 ms^2^/Hz; *P* = 0.01; Figure 3(b)) and continued to decline until the *Nrf*2^−/−^ mice were euthanized. No significant changes in LF HRV were detected in the *Nrf*2^+/+^ mice during hyperoxia. Within each genotype, no significant effect of hyperoxia on HF HRV was found, except after 35 and 36 hrs of hyperoxia when HF HRV was significantly reduced in *Nrf*2^−/−^ mice compared to *Nrf*2^+/+^ mice (group mean difference 0.71 ms^2^/Hz; *P* < 0.001 and group mean difference 0.81 ms^2^/Hz; *P* < 0.001; Figure 3(c)). Thereafter, no differences in mean HF HRV were found between genotypes. Because TP HRV is the sum of LF and HF HRV, it was not surprising to find a significant overall genotype effect during hyperoxia exposure (group mean difference 0.24 ms^2^/Hz; *P* < 0.001; Figure 3(d)). Mean TP HRV in *Nrf*2^−/−^ mice was significantly lower compared to *Nrf*2^+/+^ mice from 43 hr exposure (group mean difference 0.92 ms^2^/Hz; *P* = 0.03) to the end of exposure in *Nrf*2^−/−^ mice (45 hrs). 

## 4. Discussion

 Factors contributing to oxidative stress are widely accepted as important to the pathogenesis of cardiopulmonary diseases. Examples include inflammatory lung diseases, exposure to oxidant air pollution, and a wide range of clinical scenarios that require oxygen therapy with high fraction of inspired oxygen (FiO_2_; for example, acute respiratory distress syndrome and postmyocardial infarction patients). Understanding susceptibility mechanisms for severe oxidant stresses (such as advanced cardiopulmonary disease or high FiO_2_) or less severe changes in oxidant burden (such as air pollution exposure) is a primary public health concern. Importantly, overlap in responsible mechanisms between oxidative stress inducing exposures could partially explain reported extremes in susceptibility or resistance to adverse reactions. A prominent example is the negative effect of preexisting cardiopulmonary disease on susceptibility to adverse cardiac responses to oxidative stress and poor responses to further oxidant burden induced by oxygen therapy, all of which may operate through the same or similar mechanisms. 

In this study, we found that *Nrf2* was important in cardiac responses to a severe (hyperoxia) and moderate (UF-PM) oxidant stress. A central role for *Nrf2* in resistance to hyperoxia-induced lung injury has been described in detail [34, 35], and *Nrf2* appears to be also important in epithelial cell response to particle exposure [46], especially when combined with allergy and/or asthma [36]. Since interactions between the cardiovascular and pulmonary systems are well known, lung injury response to oxidative stress is likely to involve the heart. Moreover, an influence of oxidative stress on cardiac function has been demonstrated, especially during hypoxia [47], reperfusion injury [48], and in response to particle exposure [49]. Because *Nrf2* is established as critically important in antioxidant defense, this suggests that oxidative stress was a common component to hyperoxia and UF-PM cardiac responses in this study. 

HR responses to UF-PM exposure were statistically significant though not as severe as responses to hyperoxia (see Figures 1 and 3). Nonetheless, the changes elicited by UF-PM may have physiological relevance because the mice used were young and healthy and were otherwise not compromised. Targeted deletion of *Nrf2* exacerbated the HR responses, though the mechanism through which *Nrf2* protects against the response remains unclear. It would also be of interest to determine whether interactions exist between *Nrf2* and preexisting disease and/or age, both of which are important susceptibility factors associated with PM exposure [50–54]. 

Cardiovascular responses to particulate matter exposures have been investigated in detail ([55] for review). However, little is known about genetic susceptibility to particle exposure or which sectors of the population are most at risk. Because such a large percentage of the global population is exposed to particulate matter, this presents the potential for widespread adverse health outcomes and highlights the importance of understanding susceptibility. In this study, we found overall effects of *Nrf2* deletion on cardiac responses to UF-PM that could act through similar mechanisms that become important in a compromised host, especially since pre-existing disease is an important factor in susceptibility to UF-PM exposure [52, 54, 56]. These effects may not have manifested in this experiment since all mice were otherwise healthy, and therefore subtle responses were produced.

Previously, we reported highly significant HR responses to hyperoxia that preceded changes in pulmonary function and lung injury [10], suggesting that cardiac responses to oxidative stress may predict impending adverse pulmonary events. In the present study, we found similar HR responses to hyperoxia, and *Nrf*2^−/−^ mice were highly susceptible compared to *Nrf*2^+/+^ mice, reaching the HR end point of 250 bpm ~22 hr before *Nrf*2^+/+^ mice (Figure 2). Hyperoxia is known to cause significant lung injury, pulmonary edema and, at least in patients with acute respiratory distress syndrome, for which hyperoxia is a model, poor gas exchange leading to hypoxemia [57–59]. Although we were unable to measure blood gases during hyperoxia exposure, it is known that bradycardia can result from either hypoxemia and/or permissive hypercapnia, as a consequence of respiratory insufficiency [60–62], which may be associated with chemoreceptor activation and respiratory acidosis [60]. These mechanisms may be partially responsible for the severe bradycardia observed in this and previous studies [10] exposing mice to prolonged hyperoxia, which warrants further investigation. Decreases in HF HRV have been observed when mice were exposed to a hypoxia/hypercapnia combination, suggesting a role for autonomic nervous system (ANS) control of the heart under these conditions. In this study, we found opposing overall genotype effects (*Nrf*2^−/−^ versus *Nrf*2^+/+^) for HRV phenotypes during hyperoxia or after UF-PM exposure (decreases during hyperoxia and increases following UF-PM). While these data suggest an interaction between *Nrf2* and ANS function, the opposing effects of hyperoxia and UF-PM treatment on HRV are challenging to interpret because the correlation between changes in HRV and ANS tone is currently a matter of debate (for review [63]). Nonetheless, our data do suggest a disturbance in autonomic regulation of cardiac function during hyperoxia and after UF-PM treatment that was modulated by *Nrf2*. These HRV changes may therefore have important implications for susceptibility to adverse cardiac outcomes in response to oxidant exposure. 

However, since hypoxia and hypercapnia are unlikely to result from UF-PM exposure, *Nrf2* may act through different mechanisms compared to hyperoxia exposure. For example, *Nrf2* has been implicated in defense against cadmium-induced oxidative stress in the olfactory bulb of zebrafish [64]. Interestingly, human olfactory bulb stimulation is associated with changes in HRV [65], and UF particles have been shown to translocate from the lung to the olfactory bulb of rats [66]. Taken together, it is possible that changes in HR and HRV following UF-PM exposure in this study were partially mediated through UF-PM-induced oxidative stress effects on the olfactory bulb.

While speculative, a contributory mechanism for the observed HR and HRV responses to hyperoxia could be associated with the candidate gene thrombospondin, type 1, domain containing 4 (*ThSD4* or *AdAMTSL6*) [10]. Adamtsl6 has been reported to bind directly to fibrillin-1 (Fbn-1), initiating widespread extracellular matrix (ECM) assembly, including the myocardium [67]. Fbn-1 mediates bone morphogenetic protein-induced expression of important ECM collagens. Interestingly, absence of *Fbn-1* is associated with Marfan's syndrome [68], and over expression leads to myocardial fibrosis [69]. Moreover, changes in the myocardial ECM is associated with the development of diastolic dysfunction in heart failure, even in the short term, possibly through the renin-angiotensin-aldosterone system ([70] for review). Regulation of these genes have been linked to *Nrf2* expression levels during hyperoxia exposure in mice [71], which may explain part of the cardiac responses observed here during hyperoxia or following UF-PM exposures. Further work is required to determine the importance of changes in ECM proteins in cardiac responses to oxidative stress. 

## 5. Conclusions

 In this study, we found that severe (hyperoxia) and moderate (UF-PM) environmental oxidant stressors caused HR and HRV responses in the mouse, and targeted deletion of *Nrf2* significantly augmented the detrimental responses to these environmental oxidants. The magnitude of cardiac functional responses may have been proportional to the degree of oxidant burden during hyperoxia or after UF-PM aspiration. Understanding the mechanisms by which the myocardium defends against these stressors is critical for identifying individuals at risk, and we provide evidence that *Nrf2* may be an important determinant in defense against severe and moderate oxidative stress.

## Figures and Tables

**Table 1:** Pairwise comparisons for overall effects between three-way ANOVA factors for heart rate (HR) and heart rate variability (HRV) responses to ultrafine particulate matter (UF-PM) or saline control.

	Difference between means	Direction of difference between means	*q* value	*P* value
*HR responses to UF-PM or saline *				
Comparison: treatment within *Nrf*2^−/−^				
UF-PM versus saline	21.26 bpm	UF-PM > saline	5.27	<0.001
Comparison: genotype within UF-PM				
*Nrf*2^−/−^ versus *Nrf*2^+/+^	24.29 bpm	*Nr* *f*2^−/−^ > *Nrf*2^+/+^	6.06	<0.001
*LF HRV responses to UF-PM or saline *				
Comparisons: treatment				
saline versus UF-PM	0.02 ms^2^/Hz	Saline > UF-PM	2.79	0.048
*HF HRV responses to UF-PM or saline *				
Comparison: treatment				
UF-PM versus saline	0.13 ms^2^/Hz	UF-PM > saline	4.39	0.002
Comparison: treatment within *Nrf*2^−/−^				
UF-PM versus saline	0.37 ms^2^/Hz	UF-PM > saline	9.04	<0.001
Comparison: genotype within saline				
*Nrf*2^−/−^ versus *Nrf*2^+/+^	0.15 ms^2^/Hz	*Nr* *f*2^+/+^ > *Nrf*2^−/−^	3.32	0.019
Comparison: genotype within UF-PM				
*Nrf*2^−/−^ versus *Nrf*2^+/+^	0.33 ms^2^/Hz	*Nr* *f*2^−/−^ > *Nrf*2^+/+^	8.06	<0.001
*TP HRV responses to UF-PM or saline *				
Comparison: treatment within *Nrf*2^+/+^				
saline versus UF-PM	0.17 ms^2^/Hz	saline > UF-PM	3.78	0.007
Comparison: treatment within *Nrf*2^−/−^				
UF-PM versus saline	0.35 ms^2^/Hz	UF-PM > saline	8.22	<0.001
Comparison: genotype within saline				
*Nrf*2^−/−^ versus *Nrf*2^+/+^	0.19 ms^2^/Hz	*Nr* *f*2^+/+^ > *Nrf*2^−/−^	4.06	0.004
Comparison: genotype within UF-PM				
*Nrf*2^−/−^ versus *Nrf*2^+/+^	0.34 ms^2^/Hz	*Nr* *f*2^−/−^ > *Nrf*2^+/+^	7.93	<0.001
